# Mesolens imaging in microbiology

**DOI:** 10.1042/EBC20250033

**Published:** 2026-09-02

**Authors:** Katherine J. Baxter, Beatrice Bottura, Gail McConnell, Liam M. Rooney

**Affiliations:** 1 School of Molecular Biosciences, University of Glasgow, Glasgow, U.K.; 2 Strathclyde Institute of Pharmacy and Biomedical Sciences, University of Strathclyde, Glasgow, U.K.; 3 Department of Bacteriology, School of Infection & Immunity, University of Glasgow, Glasgow, U.K.

**Keywords:** Biofilms, Cross-scale imaging, Microbial Communities, Multi-modal imaging, Optical Microscopy

## Abstract

The optical microscope has served as an essential tool for the microbiologist since van Leeuwenhoek’s observations of ‘animalcules’, yet the core design of the objective lens has changed little over the last 100 years and imparts a dichotomy between resolution and field of view. High numerical aperture objectives resolve subcellular detail but sample only small regions, whereas low-magnification objectives capture large areas with limited resolving power. Many microbial processes span the micron-to-centimetre length scales, constraining how spatial heterogeneity, microbial interaction, and rare events can be visualised in intact specimens. This review describes how the Mesolens overcomes this limitation by combining low magnification with high numerical aperture to deliver sub-micron resolution across multi-millimetre fields of view and millimetre-scale depths. We summarise the optical principles and imaging modalities that underpin the Mesolens, including widefield and confocal laser scanning mesoscopy, and emerging implementations such as light-sheet mesoscopy, mesoscopic total internal reflection fluorescence, and standing-wave mesoscopy. We highlight applications where spatial context is essential; the discovery and characterisation of biofilm nutrient transport channels in *Escherichia coli*, understanding the architecture of cross-kingdom *Candida albicans*–*Staphylococcus aureus* biofilms, and translational applications in clinical and environmental microbiology. Finally, we discuss specimen preparation, analysis workflows, and future opportunities in new modalities, computationally guided acquisition, and scalable optical manufacturing. Together, these advances position the Mesolens as a versatile tool to resolve microbial interactions and dynamics across length scales associated with priority areas in microbiology.

## Introduction to optical mesoscopy

Optical microscopy remains one of the most versatile and informative tools in biochemistry and cell biology. From the discovery of cells to the visualisation of molecular interactions, the ability to image biological structures using light has transformed our understanding of living systems. However, a persistent challenge lies in bridging the scale between subcellular and tissue-level imaging. This spatial range is often known as the mesoscale. Traditional high numerical aperture (NA) objectives offer excellent resolution but only over small fields of view, whereas low-magnification optics can capture images of large areas, but resolving power is poor.

The development of the Mesolens addresses this long-standing trade-off. Designed to provide sub-micron resolution across a field of view (FOV) more than 4 mm wide and of specimens up to 3-mm thick, the Mesolens is an imaging objective that enables imaging of whole biological specimens, such as embryos [1], developing *Drosophilia* larvae [2], zebrafish models [3], and mature colony biofilms [4], while still resolving individual cells. To appreciate how such performance is achieved, it is useful to review the fundamental principles of optical imaging that underpin the operation of all light microscopes, including those at the mesoscale.

The Mesolens has a unique combination of a fixed low-magnification, to sample large areas, and high NA, to resolve subcellular detail. Combined, these variables break the traditional ratio between magnification and NA that limit multi-scale imaging. For example, a typical microbiologist may wish to survey their sample at low magnification using a 4×/0.10 NA objective lens and then image their chosen region of interest using a 60×/1.50 NA objective lens; both have a ratio of 1:40, precluding the ability to resolve fine spatial structures over large areas in a single acquisition. The Mesolens has a prescription of 4×/0.47 NA—a ratio of 1:8—permitting visualisation and resolution of structures on the order of 700 nm throughout a 108 mm^3^ capture volume [1,2]. Coincidentally, this prescription is ideal to sample the spatial scales over which many microbiological processes and challenges manifest, positioning the Mesolens as an emerging and powerful tool for the microbiologist.

The basic principles of light, resolution, contrast, and FOV underpin all optical imaging, from simple microscopes to advanced mesoscale systems. The Mesolens exemplifies how careful application of these principles, combined with innovative optical design, can overcome long-standing trade-offs between resolution and FOV. By facilitating subcellular-resolution imaging across multi-millimetre-scale volumes, the Mesolens bridges the gap between microscopy and macrophotography, enabling subcellular resolution imaging with the possibility of molecular specificity.

### Fundamentals of optical imaging

While light exhibits both the properties of a wave and a particle, most optical imaging systems consider only the wave nature of light. When light interacts with matter, it can be refracted, reflected, scattered, and diffracted. These processes determine how an image is formed, and what spatial information can be captured. In microscopy, the objective lens is the key element that collects and focuses light emerging from the specimen to form a magnified image.

A crucial parameter in optical design is the NA, defined as

**Figure 1:**
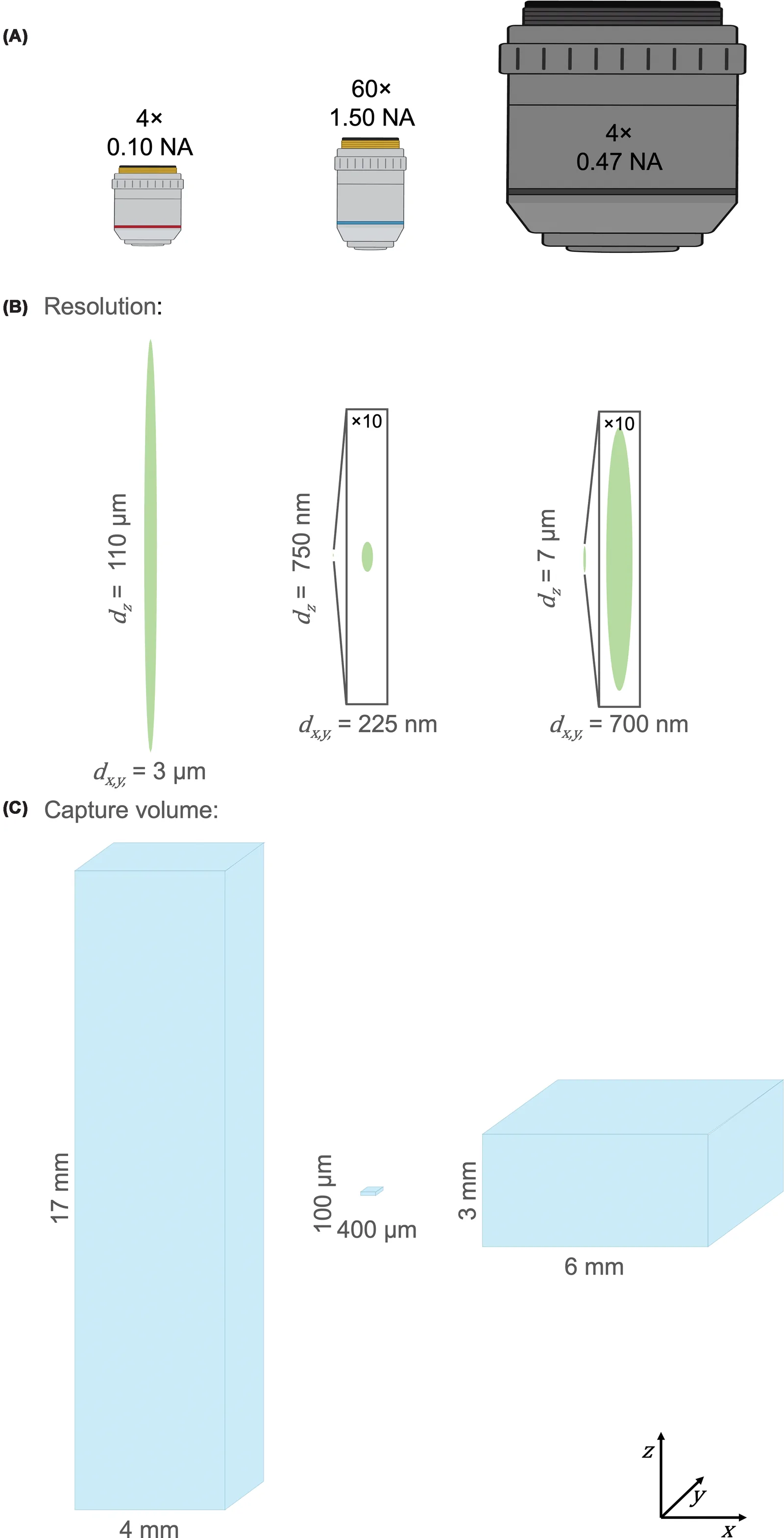
A comparison of the performance of a standard low magnification and high magnification objective lens to the Mesolens (**A**) A schematic showing the optical prescription of the three lenses. (**B**) A scaled visualisation of the point spread function (PSF) of each objective lens and attainable resolution calculated using the Rayleigh criterion, as in Equations 2 and 3. The 60× and Mesolens PSFs have been magnified by a factor of ×10 for visualisation purposes. (**C**) A scaled visualisation of the maximum capture volume, noted by the FOV size (*x, y*) and the working distance of the objective lens (*z*).

(1) NA=nsinθ,

where *n* is the refractive index of the imaging medium or specimen, and *θ* is the half-angle of the light cone collected by the objective (Figure 1A). The NA quantifies the light-collecting capacity and directly influences both image brightness and resolution [5].

To maintain image quality, optical aberrations, such as chromatic and spherical aberrations, must be minimised through precise lens design [6]. The Mesolens is distinctive in achieving diffraction-limited performance across a large FOV. More than seventeen individual lens elements are included in the prescription, with the largest lenses being more than 60 mm in diameter, and they are precisely located within the objective lens barrel to minimise optical aberrations [1]. Further reduction of optical aberrations, particularly coma, chromatic aberration, and spherical aberration, is achieved by use of correction collars [2], which can be adjusted to give diffraction-limited imaging resolution in specimens with refractive indices from *n* = 1.33 to *n* = 1.51, thus making it compatible for multi-colour imaging in three-dimensions (3D) with most biological specimens.

### Resolution

Resolution defines the smallest distance between two points that can still be distinguished as separate. The Rayleigh criterion defines the theoretical bound of lateral resolution as

(2) $$d_{x,y}=\frac{0.61\lambda }{NA},$$

where *d_x,y_* is the smallest resolvable distance and *λ* is the wavelength of light. The axial resolution, which is along the optical axis of the microscope, is given by

(3) $$d_{z}=\frac{2n\lambda }{NA^{2}}.$$

In practical terms, achieving high resolution depends not only on wavelength and NA but also on illumination uniformity, detector sampling, and system stability [5]. Traditional optical systems face an intrinsic compromise: high NA lenses are invariably high in magnification, meaning that the improved resolution can only be obtained within a tiny volume. The Mesolens uniquely circumvents this limitation through a low magnification (4×) yet exceptionally high NA (0.47) design, maintaining sub-micron resolution across a field exceeding 6 mm in diameter [1] (Figure 1). This capability allows imaging of entire biological structures at cellular detail.

### Contrast

In biological imaging, contrast determines the visibility of structures within the specimen. Without contrast, even high-resolution systems yield little meaningful information. Contrast arises from differences in absorption, scattering, refractive index, or fluorescence emission between regions [7].

Techniques such as brightfield and phase contrast microscopy enhance image visibility by exploiting these differences. In fluorescence imaging, contrast is generated by specific fluorophores that emit light at characteristic wavelengths upon excitation, enabling molecular specificity.

The Mesolens is compatible with multiple contrast mechanisms, including brightfield transmitted light, widefield epifluorescence recording, and confocal imaging. Its high NA and wide field permit both high brightness and excellent contrast over large specimen volumes, facilitating visualisation of complex biological samples.

### Field of view and depth of field

The FOV represents the spatial extent of the sample that can be imaged at once, while the depth of field (DOF) defines the axial range within which structures appear in acceptable focus [5]. Typically, increasing magnification or NA reduces the FOV and DOF, presenting a challenge for imaging large, thick specimens. The DOF can be calculated by

(4) $$DOF=\frac{\lambda (n^{2}-NA^{2}{)}^{1/2}}{NA^{2}}$$

Conventional objectives cannot easily deliver both high resolution and a large FOV because lens aberrations scale with aperture size. The Mesolens overcomes this through custom optical engineering, providing a millimetre-scale field with sub-micron resolution and a relatively long working distance. This combination allows entire specimens, such as *Drosophila* or whole mouse organs, to be imaged in a single dataset.

In 3D imaging, the axial resolution is equally important and depends on both NA and illumination geometry. Optical sectioning methods such as confocal scanning or lightsheet illumination can further improve depth discrimination, enabling high-quality volumetric reconstructions.

### Two-dimensional and three-dimensional imaging

Traditional light microscopy captures two-dimensional (2D) projections of a specimen. This is adequate for thin samples but is insufficient for thick, complex structures. To image in 3D, optical sectioning is required. By acquiring sequential focal planes, commonly referred to as a ‘z-stack’ and reconstructing them computationally, the full spatial organisation of the sample can be visualised without mechanical sectioning.

The PSF describes how a point source of light is represented in an image and governs both lateral and axial resolution. Deconvolution algorithms use the PSF to computationally correct blur and improve image sharpness.

The Mesolens supports several 3D imaging modalities. In confocal mode, optical sectioning is achieved through a pinhole that rejects out-of-focus light, improving axial resolution as in the original confocal laser scanning microscope. The lightsheet Mesolens, in contrast, uses planar illumination to excite only a thin optical section, reducing phototoxicity and acquisition time [9]. These methods enable rapid volumetric imaging of large biological specimens with cellular resolution.

### Imaging modalities underpinning the mesolens

#### Widefield Microscopy

Widefield microscopy illuminates the entire specimen simultaneously, capturing images in a single exposure. It offers high temporal resolution and is ideal for live or dynamic samples but suffers from out-of-focus blur when imaging thick specimens. The Mesolens widefield system leverages its large NA to maintain resolution across an extended field, producing detailed images of large specimens at the subcellular scale, and is compatible with brightfield transmission, darkfield illumination, and epifluorescence contrast. Illumination of specimens can be performed with a simple halogen bulb for brightfield and darkfield modes, and either an arc lamp or light-emitting diodes for excitation of fluorescence. Due to the high-resolution images across the large FOV of the Mesolens, a camera with an appropriate pixel size commensurate with the low magnification but high pixel number to capture the high-resolution detail is required for optimum performance. This was achieved at modest cost using a chip-shifting camera, which produced a 224-nm pixel size, satisfying the Nyquist–Shannon sampling criterion, across a 4.4 mm × 3.0 mm FOV [2,10]. With this camera, images can be acquired at relatively high speeds (0.55 Hz, or 1.8 seconds/image).

#### Confocal laser scanning mesoscopy

Confocal mesoscopy achieves optical sectioning by following the same principles as a confocal laser scanning microscope. A pair of galvanometer mirrors are used to produce a scanned focused laser point across the sample. Where the laser spot excites fluorescence from the specimen or reflection occurs in the specimen, causing backscattering of the incident light, the light that is emitted or reflected is collected by the Mesolens, and directed through a pinhole that is conjugate to the focal plane in the imaged specimen. This suppresses background fluorescence or reflections and enhances axial resolution. The confocal Mesolens system is slower to image specimens than widefield methods: to capture all information from a 6 mm × 6 mm FOV, with a pixel dwell time of 1 μs, each image takes 100 seconds to acquire [11].

#### Other mesolens modalities

Building on the Mesolens optical design, several additional modalities have emerged. By illuminating the specimen with a long, thin sheet of light, as is performed in lightsheet microscopy, lightsheet mesoscopy with the Mesolens can perform much faster 3D imaging of specimens than confocal laser scanning mesoscopy. Imaging speeds with the Mesolens and a lightsheet are 14 times faster than the confocal counterpart. However, specimen mounting for lightsheet mesoscopy is challenging. The specimen must be mounted in a chamber with two orthogonal optical quality windows, one suitable for introducing the illumination beam, and the other for imaging the resulting fluorescence from the specimen with the Mesolens. Moreover, the optical alignment of the optical system to produce the unusually long and thin lightsheet for compatibility with the Mesolens is amenable to misalignment, thereby reducing the practicality of this approach. Nevertheless, the Mesolens Airy lightsheet has demonstrated extended volumetric imaging over a 4.4 mm field with a lightsheet thickness of 7.8 μm over the full FOV [9].

Mesoscopic total internal reflection fluorescence (mesoTIRF) extends the principle of conventional total internal reflection (TIRF) imaging to the mesoscale regime by coupling the unique optical design of the Mesolens with an oblique, high-angle illumination geometry [12]. In standard TIRF, an evanescent electromagnetic field is generated at the interface between a high-refractive-index substrate (typically glass) and an aqueous specimen when the incident light exceeds the critical angle for TIRF. This evanescent field decays exponentially, with the thickness of the evanescent wave given by

(5) $$\delta =\frac{\lambda_{0}}{4\pi }{\left(N_{2}^{2}\sin^{2}\theta_{C}-n_{1}^{2}\right)}^{-\frac{1}{2}},$$

where *θ_c_* is the critical angle given by

(6) $$\theta_{C}=\sin^{-1}\left(\frac{n_{2}}{n_{1}}\right)$$

and *n_2_* and *n_1_* are refractive indices adjacent to a boundary, e.g. a cell-coverslip interface, and *n_2_* ≤ *n_1_*.

The approximate thickness of the evanescent wave is ∼100 nm. This results in exceptional axial confinement and high signal-to-background ratio imaging of regions of the specimen in contact with the coverslip [13]. The mesoTIRF implementation achieves the same principle but over a multi-millimetre-scale FOV, exploiting the high NA (NA = 0.47) of the Mesolens to sustain critical-angle illumination across a vastly expanded area compared to the conventional light microscope (4.4 mm versus several hundred micron). The technique thus provides a powerful means of mapping cell morphology, membrane dynamics, or focal adhesion organization across entire cultures in a single high-speed imaging acquisition.

Standing-wave mesoscale imaging applies the principles of standing-wave fluorescence microscopy to the large field and high NA of the Mesolens to achieve axial super-resolution over millimetre-scale specimen areas [14]. In standing-wave fluorescence microscopy, an interference pattern is created by the superposition of two coherent light beams, typically an incident and a reflected wave, producing a series of excitation antinodes and nodes normal to the optical axis [15,16]. Fluorophores located within the antinodes experience enhanced excitation, generating a modulated axial fluorescence pattern with an effective periodicity of approximately

(7) $$\frac{\lambda }{2n}.$$

When used in conjunction with a fluorescent membrane stain applied to cells, standing wave imaging results in a topographical contour map of the cell, encoding 3D information in a 2D image, with an axial resolution on the order of 100 nm. Unlike TIRF, the multiplanar nature of the illuminating standing wave means that specimens can be imaged at depth using this approach, rather than with a single illuminating evanescent wave that can only map the part of the cell membrane in contact with the coverslip. While the axial resolution of standing wave microscopy is higher than that of confocal microscopy by approximately a factor of 8, there are information gaps caused by the modulated illuminating beam in standing wave imaging. Multicolour imaging has been used to fill in these gaps, and in the similar label-free method interference reflection microscopy. A sampling density of 92% was achieved in the microscale, compared to a fill-factor of approximately 50% in the conventional standing wave micrographs [17,18].

Standing wave mesoscopy adapts this concept to the mesoscale by combining widefield standing-wave excitation with the high NA and high collection efficiency of the Mesolens. This configuration allows the acquisition of high-contrast contour maps of the cell membrane with a single exposure. Because the standing wave pattern is formed globally across the field rather than point-by-point, standing wave mesoscopy provides rapid optical sectioning without the need for mechanical scanning. The result is a powerful modality that yields near-isotropic resolution over large areas, bridging the gap between super-resolution microscopy and large-scale tissue imaging. In addition, the technique is compatible with multi-wavelength excitation, enabling simultaneous mapping of cell structures in three dimensions at high speeds.

To illustrate the impact of mesoscopy across microbiology, we present applications by biological context, ranging from controlled model systems to complex clinical and environmental samples, and highlight the common principles by which Mesolens imaging enables new insight across these domains.

## Mesoscopic imaging of biofilm nutrient transport channels

Biofilms are microbial communities in which the constituent cells are embedded in a self-secreted extracellular matrix. This matrix is mainly composed of water, lipids, proteins and extracellular DNA [19], and it provides structural support and protection from mechanical and chemical stress [20]. Biofilms are highly dynamic and adaptable communities that can share information and collectively process external signals [21], and for this reason they constitute both a health and an environmental risk. For example, biofilms are up to 1000× more resistant to antimicrobial compounds than their planktonic counterparts [22], and they can compromise medical and industrial equipment through contamination [23–25].

Biofilms can exhibit complex morphologies and internal patterns. The structure of the biofilm itself is affected by the properties of the growth substrate [26], and specific biofilm morphologies can result from mechanical instabilities that occur during their growth and expansion [27]. For example, differences in biofilm growth rate can lead to the formation of patterns in both 2D (e.g. irregular colony edges for *Bacillus subtilis* [28,29]) and 3D, in the form of axial buckling [30]. Complex internal patterns can also arise due to heterogeneity within the bacterial population [31,32].

Optical microscopy has been used extensively to image biofilms both *in vitro* and *in vivo* using fluorescence and absorption-based contrast methods [33–35]. The optical sectioning capabilities of confocal microscopy have enabled 3D imaging of biofilms since 1991 [36], however the physical compromise between NA and magnification of traditional microscopes had previously limited the amount of spatial information that could easily be extracted from these micrographs. Because of this trade-off, one could either image a small region of the biofilm at high magnification and high resolution, thereby losing the overall context, or image the full size of the biofilm at low magnification and low resolution, but without being able to resolve individual bacterial cells. Lightsheet microscopy can be used to improve the acquisition speed of large volumes of data, and it has been used to image industrially relevant biofilms in microfluidic devices at cellular resolution, albeit with a limited FOV measuring approximately 100 μm^2^ [37]. As discussed above, the Mesolens overcomes this limitation due to an unusual combination of NA and magnification, which allows for imaging at sub-micron resolution across whole, mature colony biofilms of up to 5 mm in diameter.

### Biofilm nutrient transport channels

#### Discovery and biochemical composition

**Figure 2:**
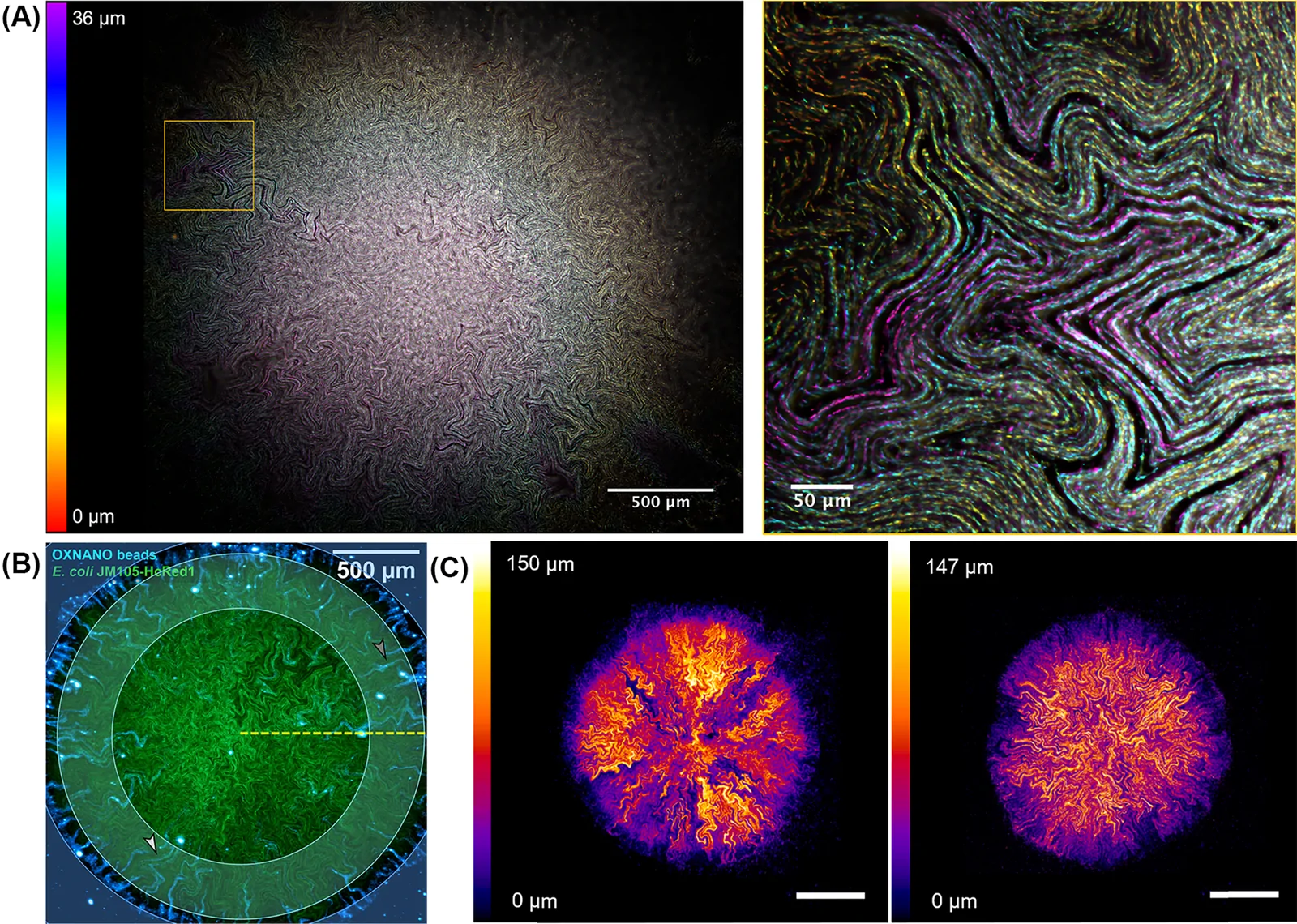
Intra-colony channels in *E. coli* biofilms (**A**) Confocal *z*-stack colour coded by depth showing the 3D distribution of channels in the biofilm volume. (**B**) Distribution of the fluorescent oxygen sensor OXNANO (cyan) within the biofilm (green). Two distinct areas (interior and exterior) are highlighted for the comparison of spatial distribution of the OXNANO signal. (**C**) Maximum intensity projection of biofilms grown on substrates with glucose limitation (15 mM carbon, left) and glucose abundance (200 mM carbon, right). Images are colour coded by depth. Scale bars 500 μm. Figures reproduced from [38,40,44].

The unique combination of low magnification and high resolution of the Mesolens enabled the discovery of a network of intra-colony channels in biofilms formed by the *Escherichia coli* K–12 strain JM105 [38]. The miniTn7 transposon system [39] was used to stably insert a photoprotein gene (*gfp* or *HcRed1* as required) into the genome of JM105, thus providing the signal for fluorescence mesoscopy. The 3D morphology of the channels was revealed through optical sectioning by confocal laser scanning mesoscopy of full, mature *E. coli* biofilms. This showed that intra-colony channels form complex, fractal-like patterns within the enclosed volume of macrocolony biofilms (Figure 2A), and they remain segregated between adjacent clonal populations. The formation of these channel networks is an emergent property of biofilm growth, as channels were observed spontaneously to reform after mechanical disruption.

Individual labelling of various structural components revealed that intra-colony channels were filled with protein-based extracellular matrix. Furthermore, channels were observed to transport small fluorescent microspheres from the surrounding environment and function as a nutrient uptake and transport mechanism, which facilitated the delivery of nutrients and key resources to the center of the biofilm. This suggests a lucrative avenue for the targeted delivery of antimicrobial compounds, which could reduce the burden of biofilms in various settings.

This was further investigated using the Mesolens, which played a crucial role in elucidating the oxygen microenvironment within biofilm transport channels. The use of a fluorescent oxygen sensor, which can be transported within the channel network, highlighted a reduction in oxygen concentration towards the centre of the biofilm compared to the edges (Figure 2B). The presence of this oxygen gradient was supported using an oxygen reporter plasmid expressing a strong fluorescent signal in the presence of anoxia, which was more intense in the centre of the biofilm [40]. This biochemical characterisation of the channel microenvironment marks an important step towards the exploitation of the channel architecture as a potential antimicrobial strategy.

#### Biofilm nutrient transport channel morphology

The chemical makeup of the growth substrate is known to influence the growth and internal morphology of bacterial biofilms, leading for example to branched morphologies in *B. subtilis* [41,42] and highly symmetric patterns in *Vibrio cholerae* [43]. The same happens for *E. coli* biofilms intra-colony channels, which expand radially outwards when grown on nutrient-deficient substrates and adopt intricate, fractal-like patterns when nutrients are abundant [44] (Figure 2C). It was initially assumed that channels were open to the surrounding environment at the biofilm edge to facilitate nutrient uptake; however, orthogonal visualisation of paraffin-embedded thin sections revealed that the channels were capped by a thin apical layer of cells. Instead, the nutrient uptake likely occurs from the base of the biofilm, where channels are often open to the growth medium containing the nutrients [40].

Biofilm fractal patterns have been observed in boundaries between isogenic strains of *E. coli* [45] and *B. subtilis* [46], where the specific geometry of the boundaries is affected by constituent cell properties such as cell shape and length [47] as well as by their metabolism [48]. In transport channels formed by the *E. coli* wide cell-shape mutant strain BW25113 Δ*ompR*::kan, the fractal complexity is affected by the chemical makeup of the growth substrate, and in particular by osmotic stress (which was investigated by altering the concentration of NaCl in the medium and by adding various amounts of iodixanol) [49].

The fractal morphology of the channels was quantified using an open-source fractal dimension image analysis package, and no relationship was established between the shape of constituent cells and the fractal complexity of the channels they formed. However, the specific *E. coli* strain used to investigate this relationship is not compatible with Mesolens imaging in water immersion (which improves image resolution compared to imaging performed in air due to the higher refractive index of the immersion fluid). The BW25113 strain used for these functional studies tended to shed under liquid immersion, which was not an issue with the original JM105 strain. For this reason, these BW25113 mutant biofilms were imaged using standard confocal laser scanning microscopy. Genetic engineering of the original JM105 strain would produce cell-shape mutants compatible with Mesolens confocal laser scanning mesoscopy in water immersion, which could provide additional 3D information and significantly increase the precision of the quantification of channel fractal morphology.

## Cross-kingdom interactions

In nature, microorganisms exist as multispecies microbial communities of prokaryotic, eukaryotic, and archaeal life within protective biofilm matrices [50]. These cross-kingdom communities are crucial for the maintenance of organism and ecosystem health, with perturbations resulting in altered stress responses, immunological dysfunction, increased likelihood of diseased states [51–53], and fundamental changes in biogeochemical processes within ecosystems [54].

Cross-kingdom biofilm development involves interspecies and intraspecies interactions, and the bidirectional exchange between the microbial community and the surrounding external milieu [50,55]. This multivariable input into biofilm synthesis allows communities to produce optimal biofilm for a given local environment, ensuring community survival under a variety of challenging growth conditions [56,57]. The inherent ability of biofilms to adapt has enabled microorganisms to colonise every ecological niche on Earth; an underpinning factor in their virulence and pathogenicity [51,58]. Therefore, understanding the cross-kingdom interactions that drive biofilm formation is a fundamental requirement to ensure ecosystem health in the face of climate change, and for the development of new drugs, tools and methodologies that can tackle rising antimicrobial resistance and improve sustainability. Mesolens imaging enables measurement and quantification of these cross-kingdom biofilm macrostructures, cellular interactions, and biofilm–surface interactions, providing new insight into cross-kingdom communities, as has been demonstrated with the *Candida* *albicans*–*Staphylococcus aureus* model community.

### Mesolens imaging of a common cross-kingdom microbial community

The pleiomorphic fungus, *C. albicans*, and Gram-positive bacterium, *S. aureus*, are two components of the skin microbiome that co-infect those with underlying health conditions and form biofilm-associated infections of indwelling medical devices [59]. Their synergism in infection occurs through reciprocal augmentation of each other’s virulence through a variety of metabolic and physical interactions [59].

**Figure 3.**
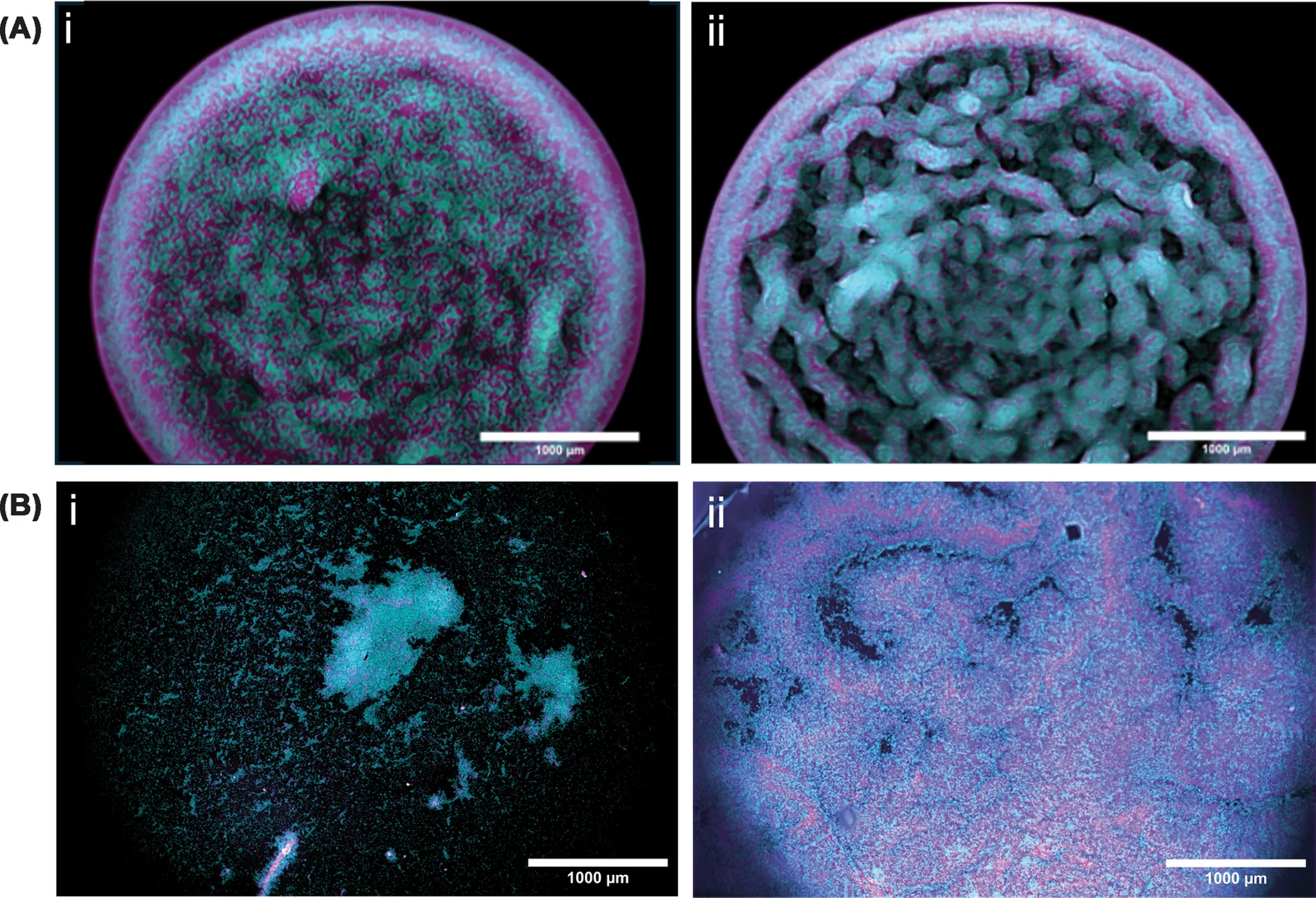
Mesolens imaging of interkingdom biofilm formation by *C. albicans* pACT1-1 GFP (cyan) and *S. aureus* N315 mCherry (magenta) (**A**) Dual species biofilms formed after 6 hours post-deposition on an agar surface. Panel (i) is formed from mixed species cultures containing yeast-form *C. albicans*, and panel (ii) from mixed species cultures containing hyphal-form *C. albicans*. (**B**) Biofilms formed on PDMS surfaces by mixed species cultured of hyphal form *C. albicans* and *S. aureus*. Panel (i) shows biofilm formation on PDMS alone, and panel (ii) is of biofilm formation on PDMS mounted on agar. Maximum intensity z projections, scale bars 1000 μm. Figures reproduced from [60,61].

Use of widefield epifluorescence mesoscopy to study *C. albicans–S. aureus* interactions has provided unparalleled visualisation of biofilm structure and provided new understandings of *C. albicans–S. aureus* dual-species biofilm formation. This approach has detailed the emergence of global biofilm macrostructures arising from deposition of the original cell population and indicated the pivotal role of *C. albicans* cell morphotype as a major structural driver of biofilm architecture [60], as can be observed in Figure 3A(i-ii), which displays an example of the two *C. albicans–S. aureu*s biofilms of differing *C. albicans* cell morphotype. In addition, the same imaging approach facilitated the study of *C. albicans–S. aureus* colonisation phenotypes on polydimethylsiloxane (PDMS), a common component of indwelling medical devices readily colonised by biofilm [61]. Mesolens imaging of *C. albicans–S. aureus* biofilm formation on PDMS in the presence and the absence of an agar tissue substitute revealed a critical role in surface colonisation and biofilm robustness for *C. albicans* hyphae (Figure 3B(i-ii). Taken together, these two Mesolens-based studies have shown that hyphae are pivotal in shaping *C. albicans–S. aureus* global biofilm formation beyond known adhesin-mediated physical interactions. Using Mesolens-based methodologies to investigate other cross-kingdom communities can address our knowledge gaps in host-biofilm dialogue, biofilm–surface interactions, and facilitate the development of biofilm-based technology platforms.

## Mesolens imaging in clinical microbiology

Clinical microbiology is inherently multiscale. Infections unfold across spatial scales, extending from subcellular host–pathogen interactions to whole-organ and systemic infections, often within specimens measuring multiple millimetres to centimetres in size. Thin tissue histology sections, cytology smears, and tissue or fluid biopsies provide high-resolution snapshots of disease, but at the cost of losing 3D context and long-range spatial relationships. Conversely, whole-organ imaging approaches routinely capture global pathology but lack the resolution required to resolve individual microorganisms or cellular interactions. This incongruity between biological scale and imaging capability presents a persistent barrier to understanding infection progression, tissue invasion, and host response.

The Mesolens addresses this gap directly by enabling subcellular resolution imaging across intact, millimetre-scale clinical specimens. Capturing large volumes in a single, continuous dataset allows host tissue, immune responses and microbial populations to be visualised simultaneously without the need for physical sectioning or computational reconstruction from tiled fields. This capability is particularly relevant for infections that exhibit spatial heterogeneity, focal invasion, or niche-specific persistence, all of which are hallmarks of chronic and systemic disease.

To illustrate the advantages of the Mesolens for clinical microbiology, we highlight applications spanning infection models, diagnostic imaging, and chronic disease, focusing on cases where mesoscopic imaging reveals spatial features that are difficult to capture using conventional approaches.

### Trypanosome-driven neuroinfection

**Figure 4:**
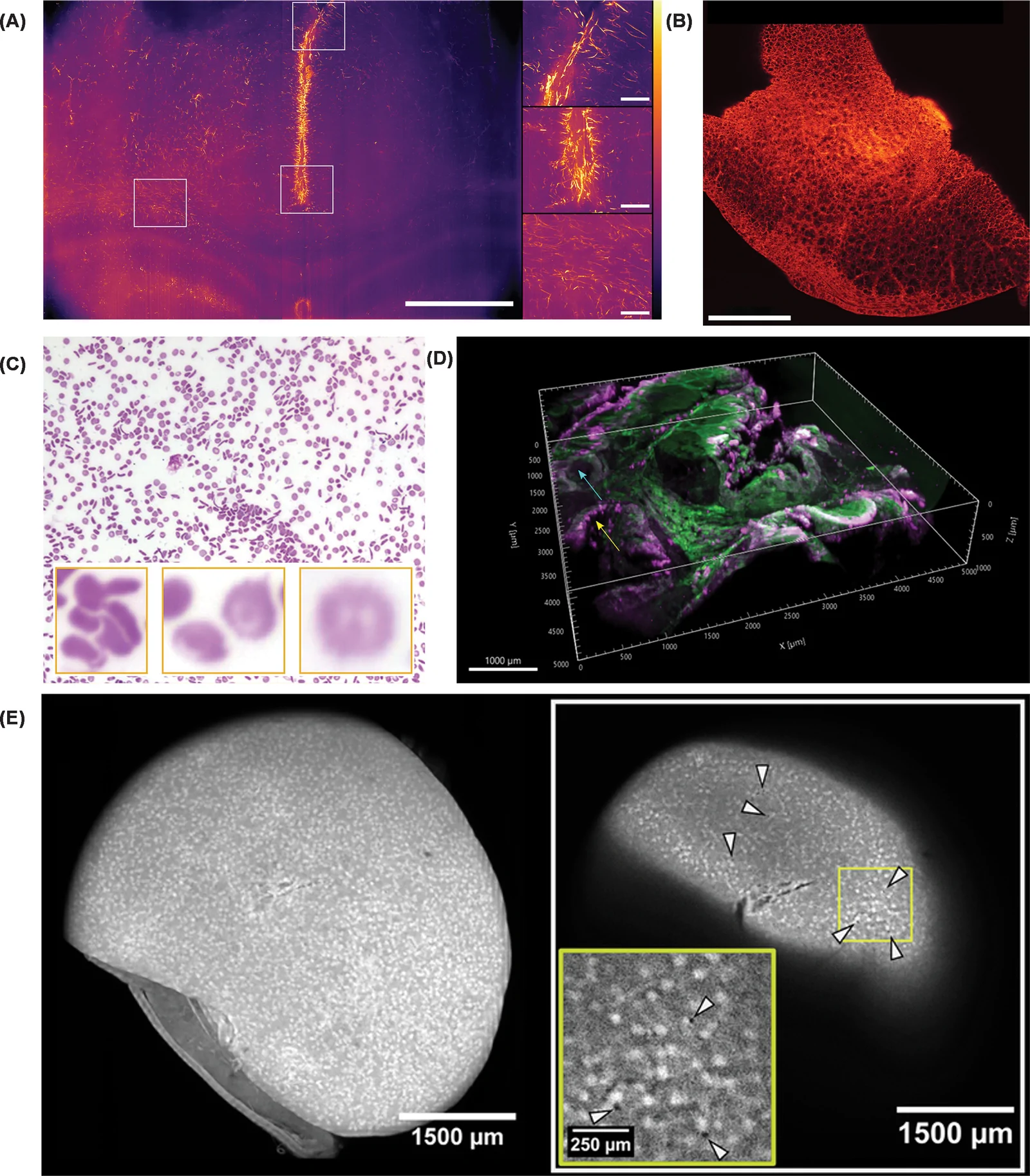
Applications of mesocopic imaging in clinical microbiology (**A**) A 3-μm thick coronal section from a *T. brucei*-infected mouse brain imaged using the Mesolens light sheet. Several regions of interest are digitally magnified to show the expression pattern of GFAP (colour coded by fluorescence intensity), a marker of astrogliosis associated with sleeping sickness. Scale bar = 1000 μm for full field, 100 μm for magnified regions. (**B**) A confocal Mesolens image of a section of intact mouse lung infected with *M. tuberculosis*. Scale bar = 1000 μm. (**C**) A full field and digitally magnified views of *Plasmodium falciparum*-infected red blood cells stained with Giemsa. (**D**) A 3D visualisation of whole-mount *ex vivo* tonsil tissue (green) and associated Gram-positive pathogens (magenta). Discrete biofilms are shown by the yellow arrow, and the opening of a tonsil crypt is shown by the cyan arrow. (**E**) A 3D render of a whole, optically cleared, kidney from an EHEC-infected mouse imaged using confocal mesoscopy. A single optical section is presented (left) with a magnified region of interest (yellow box) showing Shiga toxin-induced pores throughout the renal tissue. Figures reproduced from [11,62,72,76,79].

Mesolens imaging has provided new insight into the spatial dynamics of African trypanosomiasis, a disease characterised by systemic infection followed by central nervous system invasion [62]. Previous studies imaging trypanosome infections have typically focused on conventional histology approaches and brightfield imaging [63], low-resolution *in vivo* imaging using bioluminescent reporter strains [64,65], or *in vitro* imaging of fluorescently labelled strains [66]. These applications are limited to sampling infection scenarios in thin 2D tissue sections, or in non-representative conditions that cannot replicate infection scenarios. The Mesolens was used to study *Trypanosoma brucei* infection in a mouse model; a custom lightsheet mesoscopy approach was used to reveal the distribution of parasites, vasculature, and host immune markers across coronal brain tissue at cellular resolutions (Figure 4A). The Mesolens lightsheet setup [67] enabled visualisation of parasite accumulation along vascular networks and within specific brain regions, capturing long-range invasion pathways while simultaneously resolving individual parasites and host cells [62]. This multiscale perspective clarified how neuro-invasion correlates with vascular remodelling and immune cell recruitment, processes that are difficult to infer from conventional histological sections alone.

### Mycobacterial respiratory infection

Pulmonary infections caused by *Mycobacterium* species are defined by highly structured host–pathogen interactions, including granuloma formation and spatially segregated immune responses [68]. Despite the cross-scale nature of *Mycobacterium* infections, the host–pathogen dynamics have only been studied at the single-cell level [69], in thin 2D tissue sections [70], or at the nanoscale to understand *Mycoplasma* physiology [71]. Using the Mesolens, intact lung sections from mycobacterial infection models were imaged to reveal the organisation of bacteria, macrophages, and surrounding tissue across millimetre-scale regions [72] (Figure 4B). The resulting datasets captured entire granulomatous lesions within a single FOV while maintaining sufficient resolution to identify bacterial clusters and host cell morphology. This approach enabled direct comparison of lesion architecture across large tissue regions, providing insight into heterogeneity between neighbouring infection sites and offering a route to quantitatively assess treatment responses at the tissue scale.

### High content, machine learning-enhanced diagnostic malaria imaging

Microscopic analysis of blood films is key to diagnosing blood-borne infections, including malaria. Historically, this has been achieved using basic brightfield microscopes in the field, but recent advances in machine learning (ML) have driven innovations in smart acquisition and real-time analysis [73–75], which increase the efficiency and accuracy of clinical diagnostics. The bottleneck of detecting rare infection phenotypes across thousands of cells remains, but the capability to image throughout extended imaging volumes positions the Mesolens as a revolutionary tool for clinical diagnostics. A recent study demonstrated the application of the Mesolens in malaria diagnostics [76] (Figure 4C). *P. falciparum* parasites were detected across a population of over 13,000 red blood cells in a single acquisition, and compared to other advanced imaging, including Fourier ptychography and ML-enhanced brightfield microscopy. Clinical assessment of large cell populations in a single image, with sufficient resolving power to discern intracellular parasites, evidenced the utility of the Mesolens for quantitative imaging of rare infection events.

### Chronic bacterial infections

Chronic tonsillitis is associated with persistent bacterial biofilms embedded deep within tonsillar crypts, a spatial organisation that has hindered definitive diagnosis and treatment [77,78]. Mesolens imaging of excised tonsillar tissue demonstrated the presence, extent, and 3D organisation of bacterial communities across entire crypt structures [11] (Figure 4D). By imaging intact bulk tissue volumes rather than thin sections, the Mesolens revealed how biofilms span crypts and interface with host epithelium. These observations supported a re-evaluation of chronic tonsillitis as a biofilm-driven disease and illustrated the diagnostic value of mesoscale imaging for resolving infection niches that evade standard clinical imaging.

Enterohaemorrhagic *E. coli* (EHEC) infection causes extensive damage to the epithelial lining of the gut and, in extreme cases, remodelling of the kidney. However, these processes are challenging to assess across whole organs using conventional histology. In a recent study, the Mesolens was combined with tissue clearing to image intact mouse kidneys following EHEC infection and treatment with aurodox [79], an antivirulence compound that inhibits bacterial virulence without directly affecting growth [80–82]. Whole cleared mouse kidneys were imaged in single acquisitions, capturing millimetre-scale architecture while resolving cellular features associated with infection-induced damage and repair. Mesolens datasets revealed spatially heterogeneous remodelling of renal structures, including changes in tubular organisation and inflammatory cell distribution, and enabled direct comparison between untreated and aurodox-treated animals (Figure 4E). This approach linked antivirulence therapy to preservation of kidney architecture at the organ scale.

Together, these examples illustrate how mesoscopic imaging enables the direct observation of infection processes across spatial scales, linking cellular behaviour to tissue-level organisation. This capability provides a framework for understanding heterogeneous infection dynamics, improving diagnostic sensitivity, and evaluating therapeutic responses in intact biological systems.

### Future directions and clinical impact

The examples above illustrate how Mesolens imaging reframes clinical microbiology by sampling across spatial scales, complex architectures, and various areas of pathogen biology. Looking forward, the Mesolens is likely to expand through integration with complementary technologies. Recent advancements in multi-scale spatial transcriptomics [83–85] and highly multiplexed fluorescence labelling [86,87] could be combined with Mesolens imaging to map gene expression, microbial identity, and host responses across intact tissue volumes. Label-free modalities, such as quantitative phase imaging [88], could further reduce sample preparation complexity and increase compatibility with clinical workflows.

Equally important is the role of computation. Mesolens datasets are information-rich and well-suited to AI-driven analysis, including automated detection of infection foci, classification of tissue states, and identification of rare but clinically significant events. Smart microscopy approaches [89], where acquisition parameters are adapted in real time based on image content, could improve imaging efficiency and reduce data redundancy while preserving critical spatial information.

Taken together, the Mesolens offers a platform that supports a shift in clinical microbiology from sampling limited tissue volumes to quantifying infection and host-responses across length scales. By enabling cellular-resolution imaging across intact clinical specimens, it opens new avenues for understanding infection biology, improving diagnosis, and guiding therapeutic strategies in complex infectious diseases.

## Mesolens imaging in environmental microbiology

Microbes, in either natural or engineered environments, form complex and varied structures that span spatial scales. Soil aggregates [90–92], biofilms in aquatic systems [93,94], and microbial consortia on polymer and microplastic surfaces [95–97] can extend millimetres to centimetres [98], while the critical interactions that govern nutrient cycling, pollutant transformation, or community resilience occur at the scale of individual cells and microniches. Reconciling these scales within intact environmental samples presents a persistent imaging challenge. The traditional trade-offs between resolution and acquisition volumes that limit our ability to visualise environmentally relevant communities and phenotypes *in situ*. The Mesolens is uniquely suited to probe microbial ecology across scales.

### Soil ecology in an optically compatible 3D culture platform

Soils contain highly diverse microbial communities, where their function is tightly coupled to the physiomechanical properties and chemical gradients of their environment. However, the opacity of soil limits high-resolution optical studies due to its scattering and absorptive properties. Recent development in generating transparent soil, a synthetic, refractive index-matched 3D culture platform, has enabled optical imaging of microbial communities in mimetic soil environments [99,100]. The transparent soil system supports representative growth of soil microbes in a chemically tunable 3D environment while permitting fluorescence and transmission brightfield imaging.

**Figure 5:**
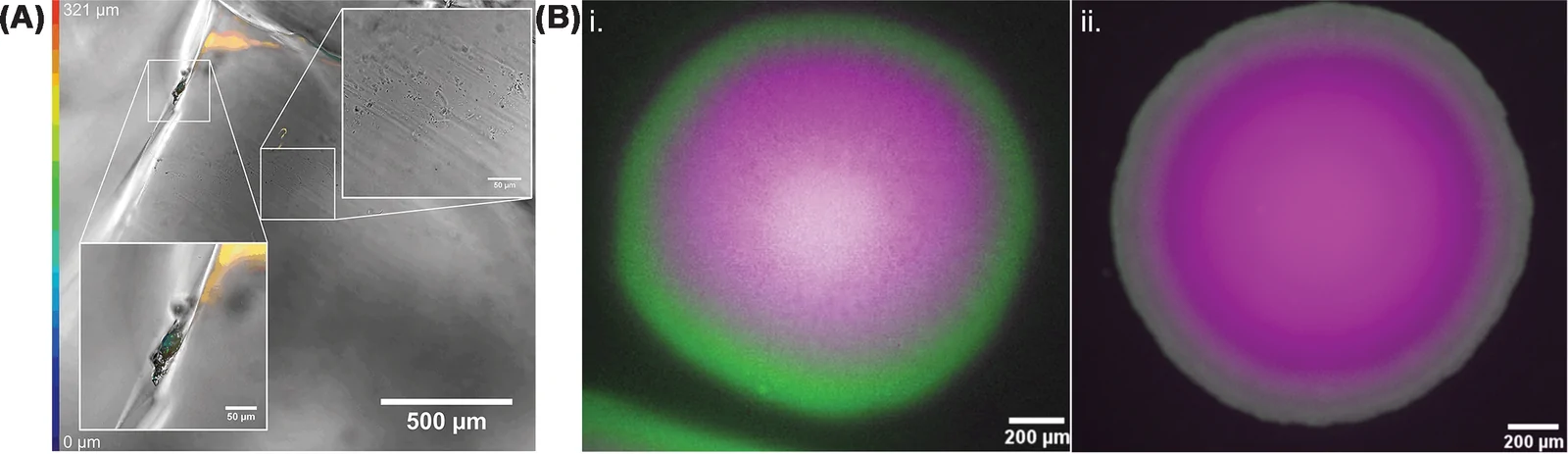
Applications of mesocopic imaging in environmental microbiology (**A**) Different colonisation patterns of *B. subtilis* on a single grain of transparent soil. The axial position of the colonising cells is shown by the colour scale, ranging over 320 μm in depth, and overlayed against a brightfield transmission image of the soil grain. (**B**) GFP-expressing *Stenotrophomonas maltophilia* biofilms under **(i)** standard condition and **(ii)** exposed to high concentrations of methylparabens. Secreted lipids were stained with Nile Red and monitored during methylparaben exposure.

Microbial colonisation and community formation in transparent soil matrices was imaged across scales using the Mesolens, revealing patterns ranging from individual cells to biofilms measuring >100 μm^3^ [101] (Figure 5A). The high resolution and acquisition volume reveal cell-to-cell and cell-to-surface interactions correlated with microhabitat features such as pore networks or soil granule shape, providing direct insight into ecological processes that were previously inferred only from indirect measurements. These datasets reveal spatial organisation patterns and community dynamics that can inform models of soil carbon turnover, nutrient fluxes, and responses to environmental perturbations.

### Physiological impact of methylparaben on drinking water biofilms

Biofilms in drinking water systems represent an interface between human health, material science, and bioremediation. Pollutants such as methylparaben, a synthetic antimicrobial preservative widely found in personal care and cosmetic products, can enter water distribution systems and influence biofilm physiology [102–104]. Traditional imaging of drinking water biofilms has been restricted to small, planar regions or thin sections [105–107], limiting interpretation of community responses across the intact biofilm.

Mesolens imaging of model *S. maltophilia* biofilms exposed to methylparaben has provided a comprehensive view of pollutant impact on biofilm structure and physiology [108]. The large FOV captures entire biofilm colonies and their internal heterogeneity, while the high resolution resolves microcolonies, extracellular matrix distribution, and disruptions to biofilm structure induced by pollutant exposure. These mesoscale datasets revealed concentration-dependent changes in biofilm architecture, spatial patterns of cell viability, and potential niches of pollutant tolerance (Figure 5B). By preserving the 3D context of these biofilms, these data suggest direct correlations between pollutant stress and ecological outcomes relevant to water quality management.

### Future directions and environmental impact

These examples illustrate how mesoscale imaging provides a flexible tool to study environmental microbiology across ecologically relevant scales. Building on these applications, the Mesolens is poised to address additional questions in environmental science. Integration with spatially resolved chemical imaging (e.g. fluorescence-labelled nutrient analogues, microelectrode arrays, or secondary ion mass spectrometry) could map metabolic activity alongside community structure within intact matrices [109–111]. Smart acquisition strategies could accelerate data capture from large, heterogeneous environmental specimens while preserving key ecological information.

The Mesolens supports a transition from fragmented, small-scale observations to holistic, multiscale characterisations of environmental microbial systems. By capturing intact volumes at cellular resolution, the Mesolens opens new opportunities to understand the spatial organisation, functional dynamics, and ecological responses of microbial communities in complex natural and engineered environments.

## Practical considerations for Mesolens imaging

### Comparisons of the Mesolens to alternative imaging approaches

Imaging large and heterogeneous microbial specimens presents a fundamental trade-off between the size of the object and the resolution attainable by the imaging system. Conventional widefield or confocal microscopy relies on stitching and tiling to reconstruct large fields of view. While effective for relatively flat samples, this approach introduces stitching artefacts, non-uniform illumination, and cumulative alignment errors, particularly in thick or optically heterogeneous tissue. Such artefacts complicate quantitative analysis and can obscure biologically meaningful gradients or boundaries.

Conventional lightsheet microscopy offers an alternative strategy, enabling rapid volumetric imaging with reduced photodamage, and has proven highly effective for cleared or optically tractable specimens [112]. However, applications of lightsheet to native biological specimens, including both clinical and environmental samples, exhibit variations in refractive index due to tissue structure, extracellular matrix composition, or particulate environments such as soil. These inhomogeneities generate shadowing, scattering, and striping artefacts in lightsheet datasets [113,114]. Optical clearing protocols can mitigate some of these effects but introduce additional complexity, potential antigen loss, and limited compatibility with routine clinical workflows [115,116].

The Mesolens mitigates several of these limitations by enabling high-NA imaging across the multi-millimetre fields of view without the need for tiling. This preserves spatial continuity and permits intact specimens to be visualised at cellular resolution, facilitating quantitative analysis of spatial organisation, heterogeneity, and rare events. In some contexts, imaging can be performed without extensive clearing or sample manipulation, maintaining biological relevance.

In addition to the Mesolens, several alternative mesoscopic imaging platforms have been developed that address the resolution-to-FOV trade-off using different optical and computational strategies. For example, the random-access two-photon mesoscopes employ nonlinear excitation over randomly selected smaller fields to achieve subcellular resolution discrete regions over large specimens [117]. This enables *in vivo* imaging of distributed neuronal populations across multiple brain regions, but not explicitly over the entire extended field at one time, as with the Mesolens. Similarly, high-speed wide-field two-photon and related systems prioritise rapid acquisition across large areas, allowing simultaneous recording of activity from thousands of cells [118], but rely on trade-offs between spatial sampling density, imaging depth, and temporal resolution

The Mesolens is optimised for dense, contiguous imaging of large specimens, preserving spatial structure and information across large volumes while maintaining subcellular resolution. It is particularly well suited to applications where preserving spatial context is essential, while alternative methods may remain preferable where temporal resolution, throughput, or accessibility are the primary considerations.

### Practical limitations and experimental considerations

Beyond the comparisons above, the practical implementation of Mesolens-based mesoscopy introduces several important constraints that influence experimental design.

#### Live imaging and temporal resolution

Live imaging is feasible using Mesolens platforms, particularly in widefield configurations, but is constrained by the acquisition speed, which is currently dictated by the readout speed of the detector. Widefield mesoscopy can achieve temporal resolutions on the order of seconds per frame, enabling observation of relatively slow biological processes. In contrast, confocal laser scanning mesoscopy is substantially slower due to point-scanning acquisition, with full-field images typically requiring tens of seconds to minutes depending on acquisition parameters. This limits its application for capturing rapid microbial dynamics with high signal-to-noise ratio. Lightsheet mesoscopy with the Mesolens has shown improved volumetric imaging speeds but introduces additional complexity in specimen mounting and optical alignment. Multi-modal acquisitions are also possible, but can introduce additional complexities of alignment, extended acquisitions, sample compatibility issues, and specimen preparation requirements; reflection and fluorescence contrast are commonly used for microbial imaging, with additional modes incorporating interference, phase and scattered light imaging.

#### Data volume and throughput

The combination of large FOV and subcellular resolution generates substantial data volumes. A single optical section can exceed 100–200 MB, and typical 3D datasets can reach tens of gigabytes depending on sampling density and specimen thickness. While such datasets are manageable for visualisation and processing using high-performance workstations, they limit the workability of longitudinal, multi-dimensional acquisitions and are not inherently suited to high-content screening workflows. However, current work focuses on implementing data reduction methods that will improve the utility of large content acquisitions.

#### Computation and data management

Mesolens datasets require careful consideration of storage, processing, and analysis pipelines. Standard image analysis tools may be insufficient for volumetric datasets of this scale, necessitating high-performance computing resources or bespoke workflows. Tasks such as segmentation and 3D quantification become computationally intensive, particularly across large volumes or multiple samples. However, processing these data types is possible using routine high-RAM workstations or high-performance computer clusters, which are becoming more accessible.

#### Instrumentation and accessibility

The Mesolens systems remain specialised and are not yet widely available, limiting accessibility and adoption across the microbiology community. There are currently four facility-grade systems in operation in the UK (three at the University of Strathclyde, Glasgow, and one at the Marine Biology Association, Plymouth), with future models in development. In addition, system alignment and optimisation require technical expertise, particularly for advanced modalities.

Combined, these factors position the Mesolens at its most effective when applied to a carefully selected biological question where multiscale spatial context is critical.

### Specimen preparation and mounting

Mesolens imaging of microbial communities requires careful specimen preparation to maintain optical quality and biological integrity. Established approaches for imaging single- and multi-species biofilms have been described previously [38,40,44,49,60], and typically rely on custom mounting strategies that enable refractive index matching and minimise optical aberrations.

**Figure 6:**
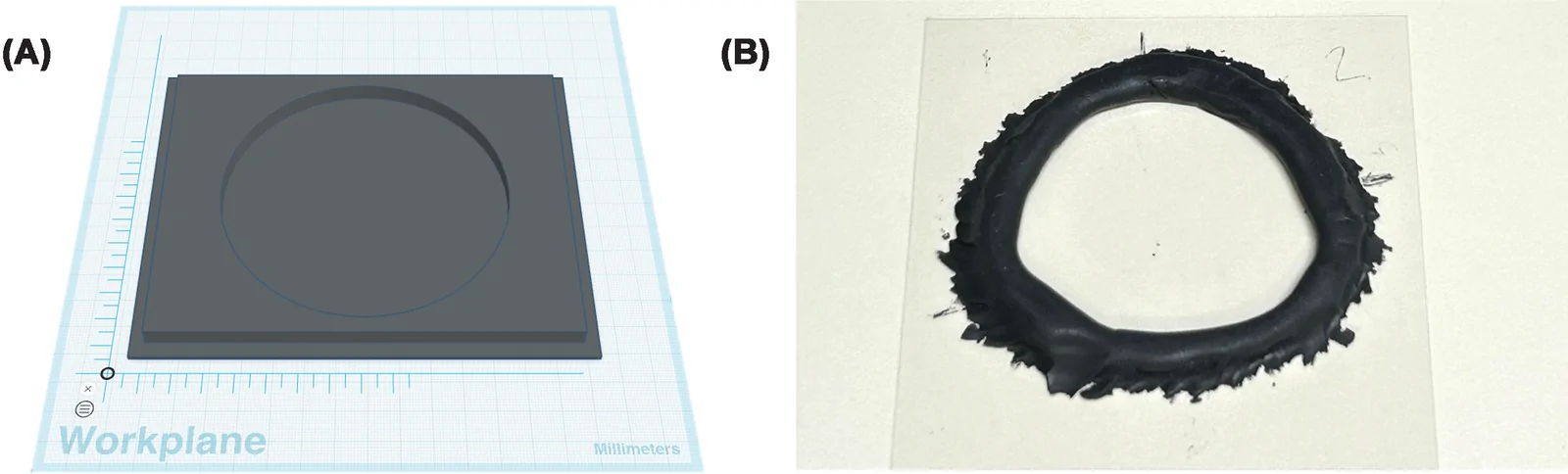
Mesolens imaging manifolds (**A**) A render of a 3D printed manifold. Used for apical imaging of biofilms. Used by Rooney et al. [38] and Bottura et al. [44,49] for imaging of single colony biofilms. (**B**) Sugru dam manifold. Used for basal imaging of biofilms, permitting time-lapse analysis of biofilm formation by Baxter et al. [60].

For macrocolony biofilms derived from single progenitor cells, a 3D-printed chamber slide containing a central agar well provides a flat imaging surface (Figure 6A). Diluted cultures are spread to yield discrete colonies, which are subsequently imaged either in air or submerged in refractive index-matched medium, depending on sample tolerance. This configuration enables imaging from the apical surface though the entire volume of the biofilm under submersion.

For colony spreading biofilms, or biofilms that are incompatible with liquid mounting (i.e. motile strains and unstable biofilm formers) an alternative basal imaging approach is used. A silicone elastomer well (Sugru) is formed on a coverslip, filled with a thin layer of agar, and inoculated with culture (Figure 6B). The coverslip is then inverted for imaging, allowing access to the biofilm-substrate interface while maintaining refractive index matching and preventing biofilm disruption.

For bulk tissue and other large-format specimens, milled anodized metal cambers have been used, which serve as both a specimen chamber and immersion bath [11]. This variety of mounting strategies demonstrate the importance of controlling sample geometry and refractive index to achieve optimal imaging performance, particularly for thick or heterogeneous specimens.

### Image analysis and computational considerations

Mesolens imaging generates information-rich datasets that enable quantitative analysis of microbial organisation and behaviour across spatial scales. Standard image analysis workflows include segmentation and feature extraction using platforms such as FIJI [119], alongside specialised tools for microbial image analysis including MicrobeJ [120] and BacStalk [121]. For biofilm-scale datasets, software such as COMSTAT [122] and BiofilmQ [123] can be used to extract morphometric parameters including colony architecture and surface roughness.

**Figure 7:**
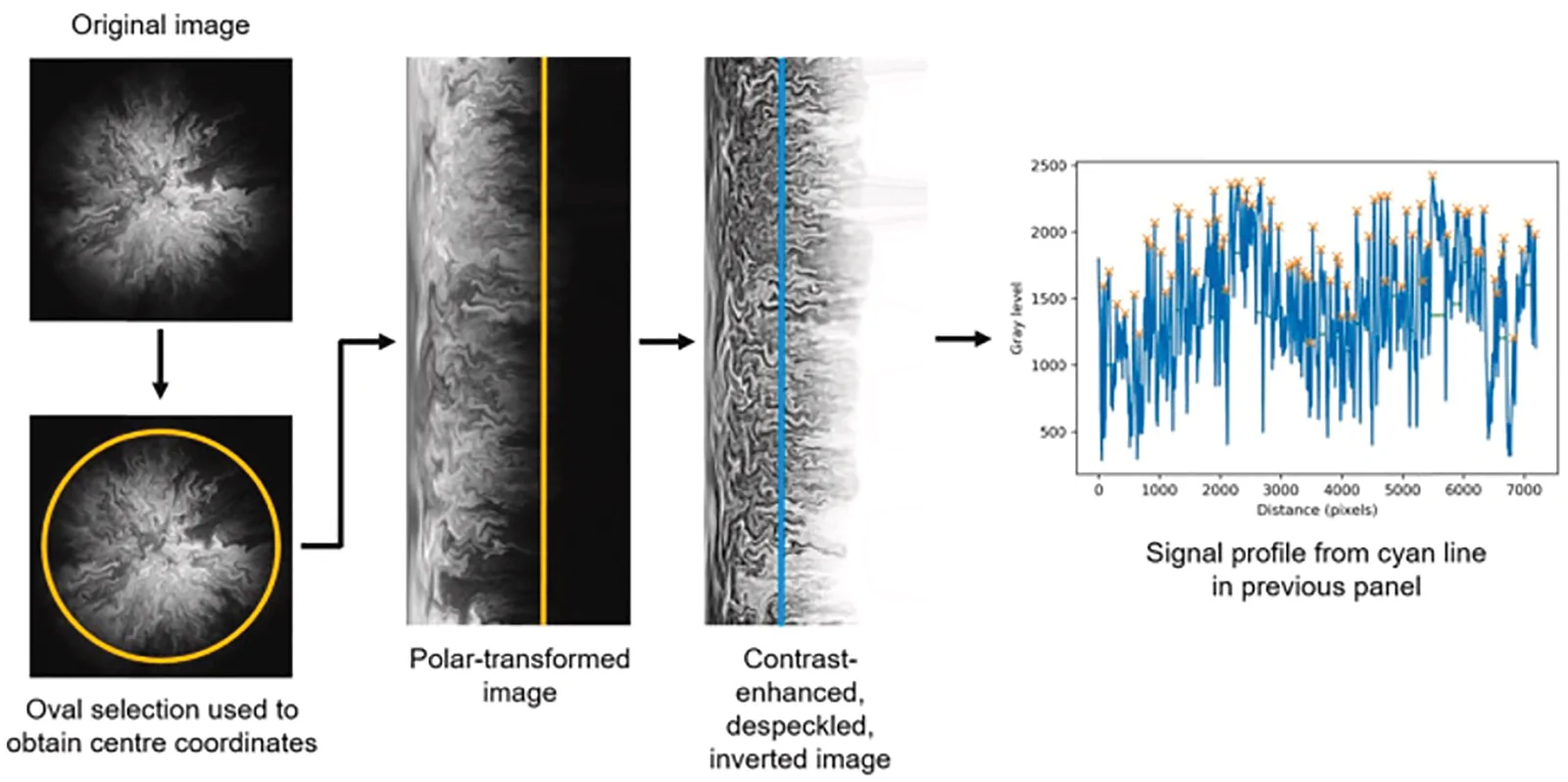
Custom image analysis pipeline used to automate measure the width of biofilm nutrient transport channels at various radial distances from the centre of the biofilm The FIJI ‘*Oval Selection*’ tool was used on the whole biofilm to obtain the centre coordinates, which were fed into the FIJI ‘*Polar Transformer*’ plugin to perform the transformation from polar coordinates to cartesian coordinates. The resulting image was contrast-enhanced, de-speckled, and inverted (to convert dark pixels bright and vice versa), and line intensity plot profiles were measured at regular intervals of 50 μm. The resulting line profile was fed into a Python pipeline that identified the peaks in the signal and calculated the full-width at half-maximum of each peak, corresponding to the channel width at that position. Figure reproduced from Bottura *et al.* [44].

However, the scale and complexity of Mesolens datasets often require bespoke analysis strategies. For example, quantification of biofilm nutrient transport channel morphology has been achieved using custom pipelines combining FIJI-based preprocessing with Python-based analysis [49] (Figure 7). Fractal analysis of routine confocal datasets from smaller regions of microcolony biofilms have revealed a strong effect of the substrate composition on the fractality of transport channels, especially when cell shape is perturbed. Fractal dimension and lacunarity can be quantified using tools such as ComsystanJ [124], although segmentation quality and image contrast can influence the accuracy of these measurements. Approaches such as relative differential box counting [125], which incorporate greyscale information, can improve robustness for low-contrast datasets. Many of these algorithms are limited in the type of digital image analysed and are often restricted to binary images. For example, the input of the FIJI plugin, twombli [126], is a binary mask of the fractal pattern that must therefore have significant contrast to be successfully segmented from the microscopy image. Many of these approaches are restricted by data volume, and present significant challenges for full-resolution multi-dimensional Mesolens data.

Recent work has begun to address the challenges of analysing large-scale mesoscopic datasets through the development of scalable, automated workflows tailored to high-resolution, large FOV imaging, including approaches that integrate ML-based segmentation and feature extraction across extended spatial domains [127]. Application of this new 3D mesoscale analysis methods has revealed subtle, but important, differences in biofilm structure that other methods have not ascertained; fractal dimension, lacunarity, entropy, homogeneity and wavelet sub-band energy, simultaneously. Furthermore, the combination of this method with timelapse imaging could help identify a mechanism for the formation of channels in *E. coli* and other bacterial species. These approaches enable automated extraction of features such as channel width across large spatial domains, reducing user bias and improving statistical robustness.

The subcellular resolution maintained in the multi-millimeter FOV of the Mesolens translates to large digital datasets: every single-colour optical section acquired at the full FOV in confocal laser scanning mode has a size of around 190 megabytes(11). Therefore, a colony biofilm with an average thickness of 250 μm can result in a 3D dataset of over 15 gigabytes (assuming slices are acquired at z intervals of 3 μm to satisfy the Nyquist–Shannon criterion). These datasets can be readily visualised with routine computers but cannot always be analysed by specialised open-source software. For example, while standard metrics such as colony biofilm area can be easily obtained in FIJI using standard segmentation tools, volumetric analysis of the biofilm morphology requires specialised software.

Overall, effective use of Mesolens data depends on integrating appropriate computational tools with experimental design, highlighting the importance of combined expertise in microscopy, image analysis, and quantitative biology.

## What is next for the Mesolens

The Mesolens has proved a powerful tool to image and quantify microbes across spatial scales, enabling subcellular resolution across intact 3D specimens that were previously fragmented by sectioning, tiling, or fixed-scale microscopy approaches. The next phase of development will likely focus on expanding the range of Mesolens modalities, alongside improving accessibility and performance through emerging optical manufacturing approaches.

From a technological perspective, continued expansion of Mesolens modalities is expected to enhance both imaging depth and speed. Advances in multi-photon imaging offer routes towards deeper volumetric imaging with reduced phototoxicity, while refinements in lightsheet mesoscopy and related illumination strategies may further improve acquisition efficiency for large specimens. Label-free modalities, including interference and reflection-based imaging, will also provide less cytotoxic and less cumbersome specimen preparation for longitudinal studies. In parallel, recent advances in 3D printed lens manufacturing and bespoke lens systems [128–132] provide a realistic pathway towards more accessible and cost-effective mesoscopes, and application-specific enhancements in aberration control, working distance and throughput.

Data-driven and adaptive imaging strategies will also improve the utility of the Mesolens for microbial imaging challenges. Current Mesolens workflows typically involve acquisitions across the entire FOV and over extended depths, generating large datasets that may contain substantial redundancy. Hierarchical or ‘smart’ acquisition approaches, where low-resolution overviews are used to identify regions of interest for targeted high-resolution imaging, or where acquisition parameters are dynamically adjusted based on image content, offer a promising route to improve efficiency while preserving critical spatial information. These strategies could enable event-triggered imaging of rare microbial behaviours, adaptive sampling of heterogeneous biofilms, and more effective use of computational resources. Integration with real-time image analysis and ML approaches would be core to these developments, enabling automated detection of phenotypes, classification of spatial patterns, and prioritisation of biologically relevant regions during acquisition.

Looking forward, the impact of Mesolens imaging in microbiology will increasingly be defined by the types of biological questions it addresses. The ability to capture intact systems at cellular resolution is particularly well suited to studying spatially heterogeneous processes, including antimicrobial resistance within structured biofilms, host–pathogen interactions across tissue volumes, and ecological interactions within complex 3D environments. Integration with multiplexed labelling, spatial ‘omics’ approaches, and chemical imaging modalities will further allow structure–function relationships to be interrogated across scales, linking microbial identity, gene expression, and metabolic activity within intact specimens.

These developments are timely; challenges in microbiology increasingly demand imaging workflows that probe deeper, faster, for longer, and smarter. Together, these developments position the Mesolens as a platform for multiscale, information-rich, integrated analysis of microbial specimens. As optical, computational, and analytical capabilities continue to converge, mesoscopic imaging is likely to play an increasing role in addressing complex biological questions that span length scales and organisational complexities.

## Summary

- Microbiology spans spatial scales, from subcellular dynamics to long-range interactions, which routine microscopes cannot adequately assess at scale with sufficient spatial resolution. The Mesolens is an optical microscope with an unusual combination of low magnification and high NA, facilitating visualisation of multi-millimeter structures with sub-micron resolution, with a range of different fluorescence and label-free imaging modalities.
- The Mesolens is emerging as a powerful tool, with demonstrations spanning basic, clinical and environmental microbiology. Modalities extend from widefield and confocal laser scanning mesoscopy, plus light-sheet mesoscopy, mesoTIRF, standing-wave mesoscopy that improve speed, optical sectioning, and axial precision for different specimen types and biological questions.
- Future innovations will likely make use of (i) smarter acquisition and AI-driven analysis designed to spot rate phenotypes and events, (ii) integration with multiplexed labelling and spatial ‘omics’ to link structure and function, and (iii) improved accessibility via scalable and lower-cost optical manufacturing.

## Open Access

Open access for this article was enabled by the participation of the University of Glasgow in an all-inclusive Read & Publish agreement with Portland Press and the Biochemical Society under an agreement with JISC.

## Abbreviations

DOF: Depth of field
EHEC: Enterohaemorrhagic *Escherichia coli*
FOV: Field of view
mesoTIRF: Mesoscopic total internal reflection fluorescence
ML: Machine learning
NA: Numerical aperture
PDMS: Polydimethylsiloxane
PSF: Point spread function
TIRF: Total internal reflection
